# Review of Sintering Methods and Polymer Materials for Powder Bed Fusion Technology

**DOI:** 10.3390/polym18202489

**Published:** 2026-10-12

**Authors:** Haihua Zhou, Ming Yang, Yanlin Song

**Affiliations:** 1 Key Laboratory of Green Printing, Institute of Chemistry, Chinese Academy of Sciences, Beijing 100190, China; seaflower@iccas.ac.cn (H.Z.); yangm04@iccas.ac.cn (M.Y.); 2 CAS Research/Education Center for Excellence in Molecular Sciences, Institute of Chemistry, Chinese Academy of Sciences, Beijing 100190, China

**Keywords:** powder bed fusion, additive manufacturing, selective laser sintering, microwave heating, high-speed sintering, multi-jet fusion

## Abstract

As a sustainable additive manufacturing method, powder bed fusion (PBF) technology has emerged as one of the important fabrication methods for mass production. The technology has several advantages, such as environmental friendliness, recyclability of materials, and being support-free, which make it have great potential in high-efficiency and green manufacturing. The sintering methods for PBF technology mainly have three technical routes: (1) conventional selective laser sintering (SLS); (2) high-speed sintering (HSS)/multi-jet fusion (MJF); (3) microwave heating (MH). This work systematically analyzes the sintering methods with their sintering characteristics and the corresponding polymer materials applied in PBF technology. The SLS method is the most widely used sintering method in PBF technology, with virtues such as cost-effectiveness, feasibility, being a robust technique and requiring no support. HSS and MJF are more efficient based on ink-jet technology. MH can assist other sintering methods with penetrating polymer powders to achieve uniform bulk heating. Commonly used polymer materials include polyamide, polypropylene, polyether ether ketone, thermoplastic polyurethane, etc. Generally, the selection of polymers is decided by the application demands. Most polymers can be recycled or reused in PBF technology. Finally, the development trends of sintering methods for PBF technology, including high-efficiency, low-energy-consumption sintering, multi-material composite sintering, and the application of new polymer materials, are prospected. This work aims to offer technical guidance for the optimization of sintering processes and the development of new polymer powder materials, thereby promoting the advancement of PBF technology.

## 1. Introduction

**Figure 1:**
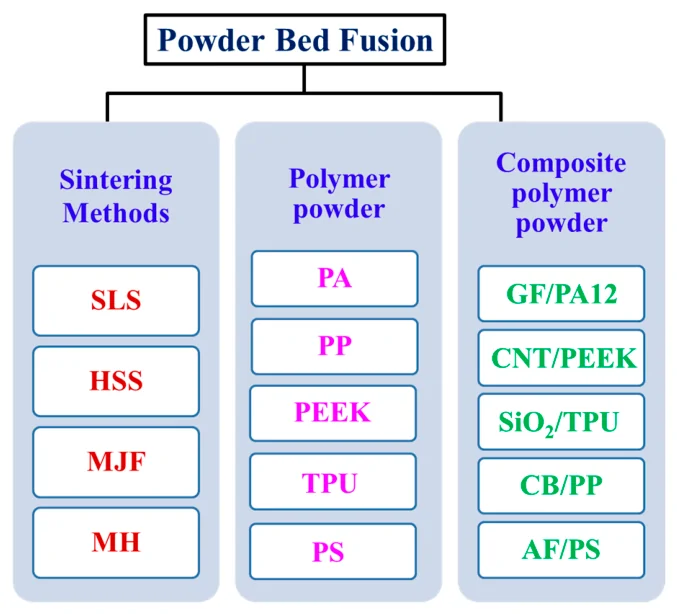
Sintering methods and polymer materials for PBF technology. Note: SLS, selective laser sintering; HSS, high-speed sintering; MJF, multi-jet fusion; MH, microwave heating; PA, polyamide; PP, polypropylene; PEEK, polyether ether ketone; TPU, thermoplastic polyurethane; PS, polystyrene; GF, glass fiber; CNT, carbon nanotube; CB, carbon black; AF, aramid fiber.

Additive manufacturing (AM) technology is developing rapidly for mass production. The global market was valued at USD 20.37 billion in 2023 and is projected to reach USD 87.2 billion by 2030. Powder bed fusion (PBF) technology is a prominent method for AM [1,2]. In order to eliminate the filamentous texture on the surface of parts fabricated by fused deposition modeling (FDM), developed by Crump of Stratasys Ltd., Minnetonka, MN, USA. Deckard proposed the selective laser sintering (SLS) technique, which was considered the origin of PBF technology. Compared with FDM, the SLS process exhibits several advantages, such as support-free manufacturing, superior mechanical isotropy, and the ability to produce highly complex geometries [3]. Since commonly used polymer powders are prone to shrinking during the SLS process, preheating the powder bed is essential prior to sintering. SLS typically employs CO_2_ lasers, which are highly effective for polymers due to high absorption rates. Consequently, laser-based methods can provide superior densification and mechanical properties compared to conventional thermal sintering [4]. Different to the SLS method, microwave heating (MH) penetrates polymer powders to achieve uniform bulk heating. When combined with suitable shape-memory polymers, MH assists the SLS process in improving sintering quality [5]. High-speed sintering (HSS) is an alternative to conventional sintering methods with low cost and high efficiency. The process begins by inkjet-printing infrared-absorbing materials onto polymer powders. Then, these materials absorb infrared energy to initiate sintering. Thus, the HSS method replaces expensive laser devices with an inkjet printer [6]. This review analyzes the characteristics of three categories of sintering methods: (1) SLS and auxiliary approaches, such as the combination of SLS with MH; (2) HSS methods based on inkjet technology; and (3) multi-jet fusion (MJF) and MH methods. Concurrently, related polymer materials, including polyamide (PA), polypropylene (PP), polyether ether ketone (PEEK), thermoplastic polyurethane (TPU), etc., are systematically investigated. Finally, development trends in sintering methods and polymer materials for PBF technology are prospected. This review will not only be helpful in selecting proper sintering methods, it will also be valuable for finding suitable polymer materials which meet diverse application requirements (as shown in Figure 1). The review will afford technical support for the optimization of sintering processes and the selection and development of related polymer powder materials.

## 2. Sintering Methods

As mentioned above, the most common PBF sintering methods for polymer materials include SLS, HSS, and MJF. Among these, the SLS process relies on precise computer-controlled laser scanning and uses polymer powders as the building materials. A three-dimensional (3D) part is fabricated by selectively sintering a cross-section of the powder bed, followed by the bonding of successive layers. The SLS process begins by uniformly spreading a thin layer of powder onto the substrate using a roller or blade. Subsequently, a laser beam selectively sinters the powder according to a predefined path. The powder particles fuse together upon laser irradiation, and the desired 3D part is ultimately formed through layer-by-layer accumulation [7]. It is well known that the SLS method is a cost-effective, feasible, and robust technique. More importantly, unlike many other AM methods, SLS does not require extensive support structures, as the unsintered powders naturally support the sintered parts during the fabrication process. However, the rapid melting behavior of polymer powders during the SLS process leads to shrinkage, warping, and deformation of the sintered products [8].

A key distinction lies in the energy interaction: SLS typically sinters powder particles (partial melting) by laser; in contrast, HSS and MJF methods introduce infrared (IR) irradiation sintering into PBF technology, primarily for polymers.

**Figure 2:**
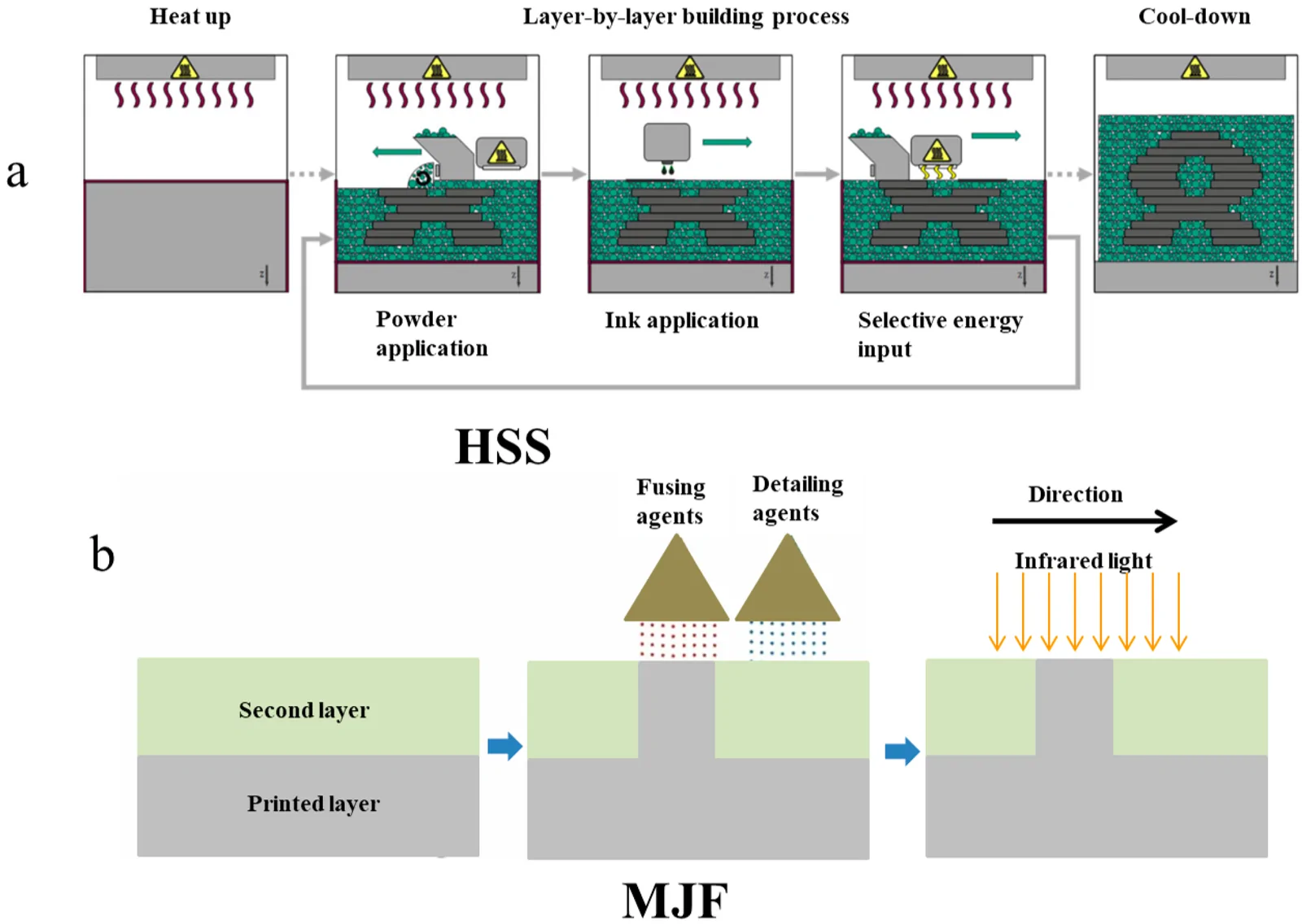
Comparison of HSS and MJF methods: (**a**) HSS (open-access article from Ref. [9]); (**b**) MJF. HSS, high-speed sintering; MJF, multi-jet fusion.

HSS utilizes inkjet printing to selectively deposit infrared-absorption ink onto a powder bed, which is sintered by an infrared lamp, while the area without ink remains unsintered [4]. MJF is essentially a variant of HSS and was commercialized by Hewlett-Packard. Unlike HSS, MJF jets both a fusing agent and a detailing agent to prevent unwanted fusion (Figure 2a,b). Furthermore, HSS employs two types of infrared lamps, one emitting near-infrared radiation for sintering and the other functioning as a mid-infrared overhead heater, while MJF utilizes lamps exclusively in the near-infrared range. The HSS method employs a single type of infrared-absorbing ink, which is selectively applied to the powder bed. It utilizes infrared lamps emitting two distinct wavelengths to sinter the material, thereby enabling the fabrication of 3D products. In contrast, the MJF method uses a single type of lamp but relies on two water-based agents: fusing agents and detailing agents. Notably, both HSS and MJF technologies utilize carbon black (CB) as the primary radiation-absorbing material. Due to their compatibility with multi-functional polymer powders, MJF and HSS have emerged as leading methods in PBF technology. Moreover, parts fabricated via these processes exhibit intricate geometries and competitive surface quality without the need for support structures [9,10,11,12,13,14].

MH has also been widely adopted as a sintering method in PBF technology. In contrast to laser or infrared light, MH provides volumetric heating by microwaves, which enables uniform sintering throughout all printed layers. This method accelerates reaction kinetics without compromising the final material properties.

Compared with conventional heating, MH has several advantages, including environmental friendliness, significantly improved interlayer bond strength, substantial energy savings, and enhanced microstructure development. MH has been primarily applied in the sintering of polymers, ceramics, and their composites. However, since neat polymers generally have low dielectric loss factors at standard microwave frequencies (2.45 GHz), the MH method requires dielectric dopants/susceptors (e.g., CNTs, ferrite, and ionic liquids) to achieve adequate coupling. At the same time, challenges such as uneven temperature distribution and low energy conversion efficiency remain to be addressed [15,16,17,18,19,20,21,22,23,24].

In addition to the sintering methods mentioned above, several other methods have emerged, including Large-Area Projection Sintering (LAPS), Selective Inhibition Sintering (SIS), ProFusion, and Single-Pass Jetting (SPJ). Specifically, LAPS adopts visible light to generate thermal energy for sintering polymer powders; SIS employs an inhibiting agent to achieve selective sintering. ProFusion melts polymer powders using multiple lasers guided by CAD models. SPJ integrates with binder jetting technology to reduce sintering time [4]. A comparison of the characteristics of SLS, HSS, MJF, MH, LAPS, SIS, ProFusion, and SPJ is presented in Table 1.

The selection of polymer materials for various sintering techniques is dictated by the specific application requirements.

## 3. Polymer Powders

The SLS method is the most prevalent technique in PBF technology. The method utilizes a laser with a specific power density to fabricate 3D parts through layer-by-layer sintering [25]. Theoretically, any polymer powder capable of solidifying or melting without thermal decomposition during the SLS process is suitable for PBF technology. However, only a limited range of polymers meet the requirements for commercial application. The polymers discussed herein include PA, PP, PEEK, and TPU, as well as others such as polystyrene (PS), polycarbonate (PC), ultra-high-molecular-weight polyethylene (UHMWPE), and ethylene vinyl acetate copolymer (EVA) [26,27,28,29,30].

### 3.1. PA

As an engineering polymer, PA, commonly known as nylon, is widely used in PBF fabrication. It exhibits excellent mechanical properties, including high tensile strength, superior impact resistance, exceptional processability, low moisture absorption, and rapid melting kinetics. Furthermore, the biocompatibility of PA renders it highly attractive for biomedical applications [31,32,33,34]. Current research on PA primarily focuses on particle size, crystallization behavior, aging mechanisms, composite PA materials development, and the influence of laser power density, which are vital for the resulting parts during the sintering process [35,36,37,38,39].

#### 3.1.1. Particle Size

**Figure 3:**
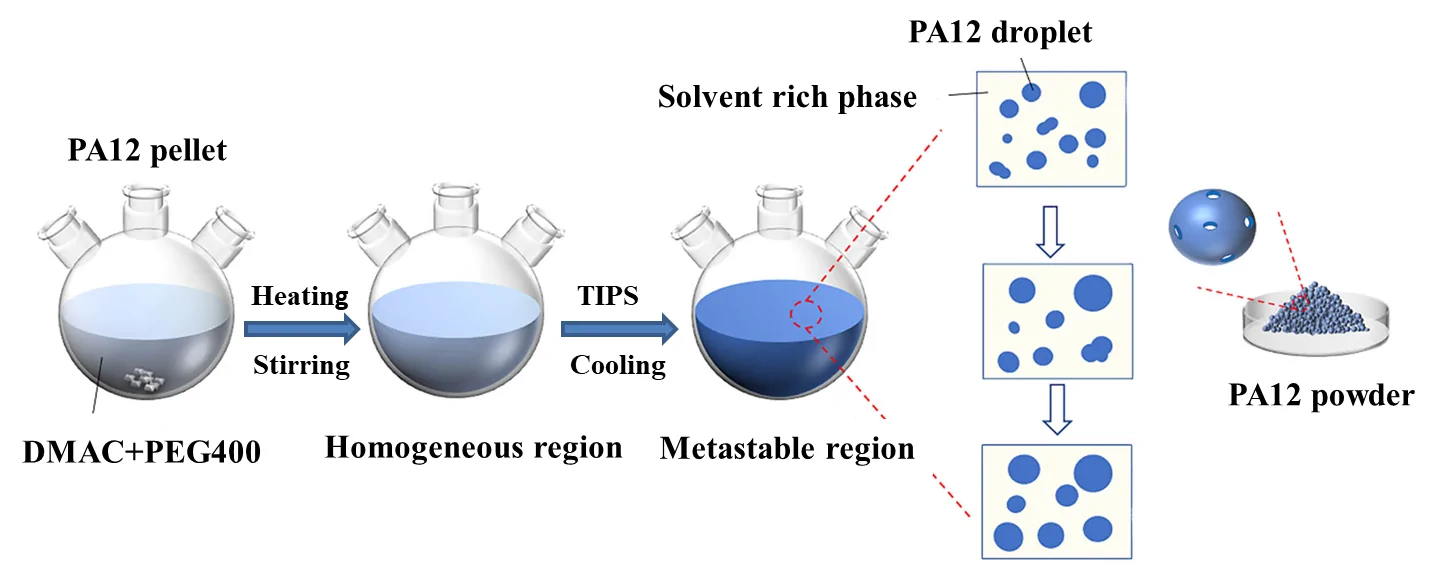
Schematic of PA12 powders prepared by thermally induced phase separation method assisted by N, N-dimethylacetamide (DMAC) and polyethylene glycol 400 (PEG400); reprinted with permission from Ref. [36], copyright 2022, De Gruyter.

Particle size is determined by a fabrication method using PA powders. For instance, PA powders produced via mechanical crushing typically exhibit poor flowability due to their irregular morphology and broad particle size distribution [40]. In order to better control the particle size, Jin et al. employed solvent precipitation to prepare PA1212 with a particle size range of 20–80 μm at a specific temperature. The resulting PA powders demonstrated superior bending strength and elastic modulus compared to the commercial counterparts [41]. Additionally, Zou et al. synthesized polyamide 6-polyurethane copolymer powder using anionic suspension polymerization. They enhanced the compatibilizing effect with a high PU content in the copolymer. Both the strength and toughness of the powders were improved [42]. Su et al. used a thermally induced phase separation (TIPS) method to prepare PA12 powders (Figure 3). The resulting powders exhibited an average particle size of 55.2 μm with a narrow size distribution. Furthermore, their spherical morphology ensured excellent flowability and enhanced crystallinity [36]. Similarly, Tischer et al. fabricated PA11 powders using the same TIPS method, incorporating SiO_2_, Al_2_O_3_, TiO_2_, or magnetic iron oxide into the particles during the process. They observed that the composite PA11 powders containing SiO_2_, Al_2_O_3_, or TiO_2_ maintained good flowability even at elevated temperatures, without compromising the thermal stability or crystal structure of the PA11 matrix. The influence of the oxide additives on thermal properties takes the following order: TiO_2_ > Al_2_O_3_ > SiO_2_ [43,44]. Differential scanning calorimetry (DSC) is widely employed to gain a deeper understanding of the crystallization process in PBF technology [45]. Generally, a single melting peak suggests the presence of a single crystal form. More significantly, DSC can be employed to determine the width of the sintering window, defined as the temperature range between the onset of melting and the onset of crystallization. By using DSC measurements, the authors found that the crystallization of PA11 was affected by the type and content of the additives. And the additives contributed smaller sinter windows and shorter crystallization times. In Table 2, a comparison of different preparation methods and PA particle sizes can be seen. The resulting powders differ in terms of composition and morphology. Furthermore, PA11 composites reinforced with hollow ceramic microspheres composed of SiO_2_, Al_2_O_3_, Fe_2_O_3_, and TiO_2_ achieve lightweight structures with enhanced wear resistance [46].

#### 3.1.2. Crystallization of PA

As is known, crystallization significantly influences the mechanical and thermal properties of sintered PA parts. In PBF technology, semicrystalline PA is prone to shrinkage during the sintering process. Consequently, understanding PA crystallization kinetics has become a key research focus for controlling the degree of crystallinity. Crystallization kinetics research primarily focuses on isothermal and non-isothermal crystallization processes. For instance, Zhao et al. observed consistent trends in both isothermal and non-isothermal crystallization process: a higher isothermal holding temperature resulted in a longer crystallization time. Further, a higher initial temperature required a longer crystallization time due to the associated slower cooling rate. The degree of crystallinity was determined using the Avrami model, while models for non-isothermal crystallization were established based on the Ozawa, Jeziorny, and Nakamura equations [47].

Teo et al. demonstrated that both crystallization time and the cooling rate influence crystal formation, phase composition, and shrinkage. They proposed that the effect on the crystal formation process started with the nucleation of α’ crystals upon cooling. Then, the intensity at the (001) crystal plane increases with decreasing temperature. Finally, γ crystals are formed under faster cooling conditions. Additionally, the study also revealed that crystal shrinkage was correlated with lamellar folding activity and intensified with increased cooling rate [48].

In addition to isothermal/non-isothermal conditions and cooling rates, crystallization kinetics can be modulated by incorporating additives such as oxide nanoparticles (SiO_2_, Al_2_O_3_, and TiO_2_), magnetic nanoparticles, and carbon nanoparticles (CNPs). Tischer et al. incorporated SiO_2_, Al_2_O_3_, TiO_2_ or magnetic iron oxide into PA12 and found that all these additives accelerated crystallization and reduced the crystal particle size [43,44]. Similarly, Sommereyns et al. introduced CNPs into PA12 powders and observed improvements in light absorptivity (particularly in visible and infrared light), thermal stability, and electrical conductivity. Concomitantly, the CNPs evidently altered the crystal structure. Notably, the morphology of PA12 crystals transitioned from spherical to ellipsoidal with increasing CNP content [49].

#### 3.1.3. Aging of PA

During the SLS process, PA powders are subjected to elevated temperatures which make thermal aging inevitable. Although a nitrogen atmosphere is employed to mitigate oxidation, aging still significantly impacts powder properties, stability, and the mechanical performance of the final parts [50,51,52,53].

Dadbakhsh et al. investigated the effects of aging on the crystallinity, microstructure, and mechanical properties of sintered PA12 products by altering their thermal and coalescence behaviors during the SLS process. Virgin PA12 powders exhibit an approximately spherical morphology with a slightly rough surface. In contrast, aged PA12 powders develop increased cracking under high temperatures. The cracks are attributed to the evaporation of residual solvent or moisture, as well as expansion and shrinkage phenomena that occur during SLS. Virgin PA12 powders have higher crystallinity with fibrillar lamellae in spherulites, whereas aged powders display lower crystallinity with lamellae embedded in amorphous regions, resulting in reduced stiffness and strength. Furthermore, the sintering surface of virgin PA12 exhibits significant porosities which are due to the rapid formation of locally distributed, highly crystalline spherulites. In contrast, when virgin and aged powders are mixed together, the broadened crystallization interval allows sufficient time for pore filling. Parts sintered from aged PA powders have a higher density of crystallization nuclei, which have a lower surface porosity than those sintered from virgin powders. A comparison of the three types of PA powders reveals that parts produced from mixed powders demonstrate a favorable balance of tensile strength and ductility. And parts sintered from virgin PA powders have higher tensile strength and shear stress than those sintered from aged powders [54].

Stiller et al. employed machine aging for PA12 without powder refreshing and reused the aged PA12 powders for nine consecutive cycles under constant sintering parameters. They found that bulk density decreased significantly due to powder agglomeration. In addition, powder viscosity increased with rising molecular weight, while the ductility of the sintered products improved as the porosity increased [37,55].

Similar to Stiller’s work, numerous studies have been conducted on PA powder aging, as summarized in Table 3. Aging under different conditions such as PA type, atmosphere, or build cycle will affect powder particle size distribution, crystallinity, melting peak temperature, and tensile strength. Specifically, the particle size distribution of aged PA12 shifts due to the coalescence of fine particles, which is driven by elevated temperatures and high-energy laser exposure. The particle size distribution affects the flowability of powders. Poor flowability leads to a coarse surface on the sintered parts. The evolution of crystallinity in aged PA is governed by the crystallization onset temperature and crystallization enthalpy. Meanwhile, the melting peak temperature is determined by the reorganization of PA molecular chains and molecular-weight variations during the aging process. And mechanical properties such as Young’s modulus, tensile strength, and elongation at break are dictated by the content of small-sized crystals, interlayer heating during the SLS process, increased viscosity, and the extent of agglomerates. The orange-peel surface defect arises from particle cracking or the aggregation of particles with diverse morphologies, such as potato-shaped, elongated, and near-spherical forms. Porosity results from voids created by density discrepancies between sintered and non-sintered regions within recycled powders.

#### 3.1.4. Recycling/Reuse of PA Materials

PA materials used in PBF can be recycled in many ways, including blending with virgin powder (refreshing), surface modification, and chain scission of virgin powder [69]. Recently, PA powders have been recycled through chemical methods such as acid-catalyzed hydrolysis and hydrothermal treatment. Salimi et al. chemically recycled heavily used PA11 in two ways, by sulfuric acid treatment and by N_2_-assisted hydrothermal treatment. They found that the chemical treatment restored the weight-average molecular weight and polydispersity, bringing them close to those of virgin PA11. Moreover, the recycled PA11 powder was successfully reused in the PBF process. The Young’s modulus of the resulting PA11 products was better than that of the virgin ones, while the elongation only retained 48% [70].

**Figure 4:**
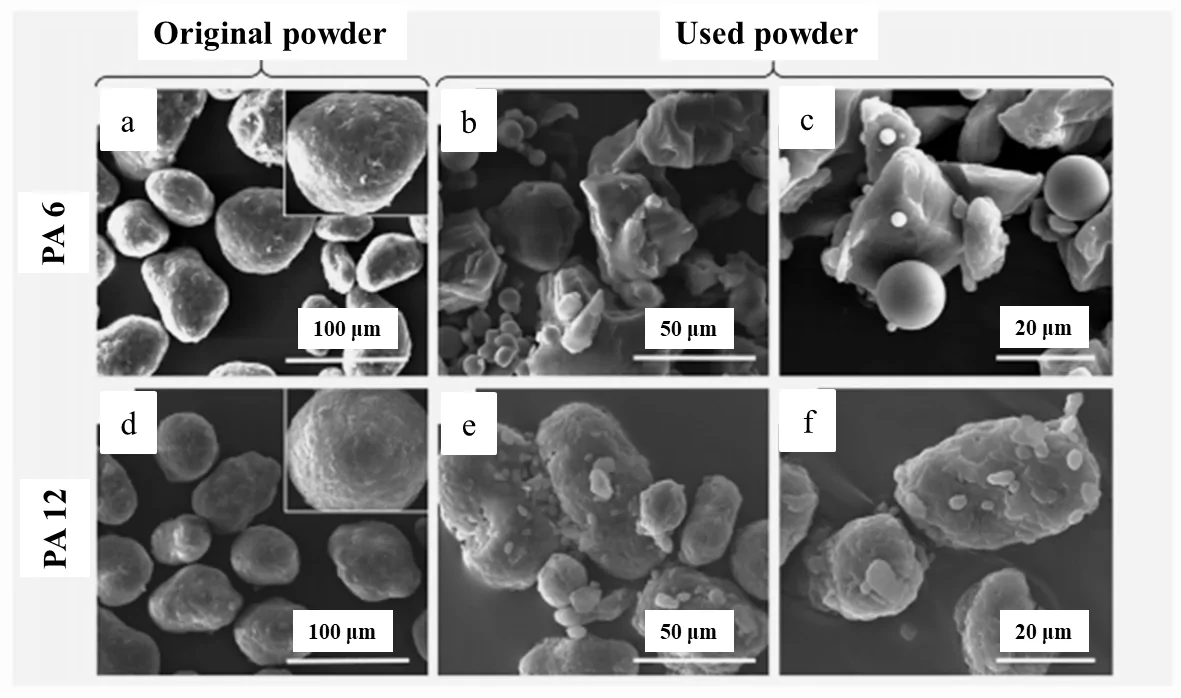
Differences in the morphology of original and used PA 6 and PA 12 powders. SEM images of (**a**) PA6, Original powder (100 μm), (**b**) PA6, Used powder (50 μm), (**c**) PA6, Used powder (20 μm), (**d**) PA12, Original powder (100 μm), (**e**) PA12, Used powder (50 μm), (**f**) PA12, Used powder (20 μm). Reprinted with permission from Ref. [72], copyright 2022 MDPI.

Radiation spheroidization was used by Gruber et al. to recycle PA12 powders. They dispersed the powders in a stream to form aerosols. Then, the aerosols were irradiated by an infrared lamp. The resultant PA powders had a near-spherical shape to ensure suitable spreading, enabling the reuse of PA6 and PA12 (as shown in Figure 4) [71,72].

#### 3.1.5. Composite PA Materials

PA6 and PA12 are the most commonly used PA materials in PBF technology. However, PA6 is highly hygroscopic, which can compromise its long-term stability and service life. In contrast, PA12 exhibits anisotropy, which will result in poor mechanical performance in the vertical build direction. This limitation can be effectively mitigated by incorporating appropriate composite reinforcements [73,74]. Recent studies indicate that the application of composite PA materials has expanded into specialized fields, including flame-retardant systems and biomedical devices [75,76,77].

Typical additives for PA6 composites include carbon nanotubes (CNTs), carbon fiber (CF), and glass fiber (GF) [78,79,80]. CNTs and CF can alleviate the adverse effects of moisture absorption on PA6 by forming a special barrier structure that inhibits water penetration. Meanwhile, these fillers also enhance the thermal and dimensional stability of PA6. GF/PA6 composites possess excellent mechanical strength and outstanding wear resistance. However, these properties depend on several factors, including a GF concentration of 30–40 wt%, appropriate extrusion and processing temperatures, and 3D printing speeds. Despite these advantages, the biomedical application of GF/PA6 composites is restricted due to the poor biocompatibility with GF [81].

For PA12, anisotropy reduces its tensile strength, elastic modulus, and fracture strength in the MJF fabrication process [82]. In order to mitigate these limitations and improve mechanical properties, various fillers have been incorporated into PA12, including CNTs, CF, GF, and oxides such as SiO_2_, Al_2_O_3_, and TiO_2_, which are similar to the additives adopted in PA11, as mentioned earlier. The incorporation of CNTs not only reinforces the mechanical strength of PA12 but also improves its electrical conductivity. Notably, CNTs facilitate the formation of a segregated conductive network even at a low loading level (less than 0.31 vol%) in PA6/PA12 composites [83,84]. Zhang et al. fabricated CF/PA12 composites by post-processing techniques (thermal annealing and microwave irradiation). These treatments resulted in a 19.34% increase in the tensile strength and a 27.11% increase in the flexural strength of the 3D-printed products [85].

GF and glass beads (GBs) are also incorporated into PA12 matrices to meet specific application requirements. For instance, GF/PA12 composites exhibit significantly higher tensile strength and elastic modulus compared to pure PA12. Furthermore, modifying the GF in GF/PA12 composites with layered double hydroxide (LDH) significantly enhanced both mechanical properties (including tensile strength, Young’s modulus, flexural strength, and flexural modulus) and flame retardancy [86,87]. Recently, PA12 blended with ammonium polyphosphate (APP), pentaerythritol (PER), and manganese (II) oxide (as a catalyst) has exhibited excellent flame retardancy [88]. Wang et al. developed PVP-SiC-MOF/PA12 composites, which demonstrate superior mechanical properties, thermal stability, and fire resistance after being disposed of by SLS [89].

Coskun et al. incorporated aminosilane-modified GBs into PA12. They demonstrated that while this modification reduced the ultimate tensile strength and flexural modulus, it significantly improved impact resistance [39]. In another study, Vindokurov et al. introduced GBs or short GF into PA12. Their findings indicated that the addition of GBs enhanced stiffness and elastic modulus, whereas the incorporation of short GF resulted in decreased tensile strength [90].

Beyond the previously mentioned investigation results, the incorporation of SiO_2_, TiO_2_, and ZnO into PA12 can enhance properties such as the laser absorbance, photocatalytic activity, and mechanical properties of sintered materials. Meanwhile, the introduction of Al_2_O_3_ into PA12 contributes to both mechanical properties and flame retardancy. SiO_2_/PA12 composites demonstrate improved laser absorbance, cost-effectiveness, and higher tensile strength. And the incorporation amount of nano-SiO_2_ has a significant impact on the sphericity and particle size of the composite powders. Specifically, SiO_2_ acts as a nucleating agent that promotes the crystallization of the PA12, thereby increasing both the crystallization temperature and the crystallinity of the nano-SiO_2_/PA12 composite. The optimal nano-SiO_2_ content was found to be 0.3 wt%, at which point the composites achieved the highest crystallinity (48.56%) and favorable flowability [91,92].

TiO_2_ serves as an excellent reinforcing component for PA12, endowing the composite with photocatalytic activity, antibacterial performance, photoelectric conversion, and self-cleaning ability. It improves the hydrophilicity, electrical conductivity, membrane performance, antifouling properties, and chlorine resistance of PA12 through effective stress transfer at the interface [93,94,95,96,97,98,99]. Afshari et al. investigated TiO_2_/PA12 powders sintered by the SLS method and found that laser power was a critical factor influencing the product quality. They considered a laser power range of 10 to 15 W to improve tensile and impact strengths, whereas powers of 15–20 W induce thermal degradation, thereby compromising mechanical strength. Additionally, increasing the laser scanning speed from 2000 to 3000 mm/s resulted in decreased mechanical strength due to insufficient heat input [94]. An et al. studied a ZnO/PA12 composite in combination with the MJF technique. In detail, they employed ZnO nanorods modified with poly (diallyldimethylammonium chloride). This surface modification facilitated uniform dispersion and effectively reduced the agglomeration of nanofillers, which resulted in improved mechanical properties [100].

The incorporation of Al_2_O_3_ or boron nitride (BN) into PA12 can significantly enhance its flame-retardant ability. Tiwari et al. incorporated 10 wt% Al_2_O_3_ nanoparticles (with an average size of 0.46 μm) into a PA12 matrix using pressureless casting. The results showed that the addition of Al_2_O_3_ has little effect on the melting temperature, but it increased the melting enthalpy and crystallinity of the composite. Concomitantly, Al_2_O_3_ effectively improved the tensile strength, elastic modulus, bending strength, bending modulus, impact strength, and density of the composite powders. These composites are expected to mitigate curling and warpage issues [101]. Furthermore, thermal conductivity can be improved by the introduction of BN into PA composites [102].

Although PA maintains a dominant position in the market, it fails to meet certain application requirements, such as chemical resistance and high impact strength. Consequently, PP has emerged as a viable alternative polymer material for PBF technology.

### 3.2. PP

As a cost-effective plastic, PP possesses several advantages, including excellent chemical resistance, low density, and low processing temperatures. Now, PP can be applied in the automotive industry, electronic instrument industry, and textile industry. It is compatible with both SLS and MJF fabrication methods. The application of PP is influenced by its polymerization type and molecular configuration. To enhance the performance of PP products, a common strategy involves incorporating nanomaterials to form high-performance composite materials [103,104,105].

#### 3.2.1. Effect of Polymerization Types

Polymerization is primarily categorized into homopolymerization and copolymerization. Nowadays, PP is commonly used in the form of isotactic PP (iPP) homopolymer, which remains a prominent subject of research [106,107,108].

Tan et al. investigated the sintering performance of iPP and copolymer PP (CoPP) during the SLS process, with PA12 as a reference (Table 4). From the Hausner ratios in Table 3, it can be observed that both iPP and CoPP have poorer flowability compared to PA12. Average surface roughness (Sa), mean height (Rc), and mean width (Rsm) indicate a coarser powder surface than that of PA12. Thus, the order of surface roughness of iPP, CoPP, and PA12 powders is CoPP > iPP > PA12. The sintering window is crucial for successful fabrication: a wider sintering window is generally preferred, as a narrow one is sensitive to temperature fluctuations and prone to be affected by negative cooling effects [106,109,110]. However, an excessively wide melting range can result in less homogeneous sintering during the SLS process. For instance, CoPP demonstrates poor resolution, is prone to caking, and is difficult to recycle.

Tan et al. also observed that a wider stable sintering window (iPP: 99 °C; CoPP: 148 °C; PA12: 162 °C) correlates with reduced polymer degradation. Moreover, a higher degree of polymer crystallinity is associated with slower cooling rates, which facilitate the formation of thicker lamellae with fewer defects. And polymer powders with lower viscosity exhibit higher coalescence rates.

#### 3.2.2. Other Influence Factors

Besides polymerization type, the sintering behavior of PP is also affected by crystallization dynamics and PP powder preparation methods. Pomatto et al. investigated the crystallization behavior of iPP under a rapid heating and cooling process. Using fast scanning calorimetry, they examined the effects of nucleation density and cooling rate on PP crystallization. Their results indicated that partial melting and nucleation density are critical for crystal morphology. Specifically, self-seeding was found to reduce the crystallization rate and caused shrinkage during the sintering process. Additionally, when the cooling rate exceeded 40 K/s, a transition from α polymorphic crystallization to mesomorphic crystallization was observed [112].

Gao et al. mitigated shrinkage and warpage of iPP-based PBF parts by optimizing printing parameters and the dosage of nucleating agents. They found that a higher laser scanning speed reduced the amount of melted iPP powder and lowered the residual stress. Both α-nucleating agents (Hyperform^®^ HPN-20E) and β-nucleating agents (WBG-II) induced more deformation than neat iPP. They attributed this phenomenon to the narrow sintering window and elevated onset crystallization temperature, rather than variations in crystallinity or crystalline structure [113].

Theoretically, low-isotacticity PP with a lower melting temperature and crystallinity can alleviate deformation by a lower powder bed temperature (Tb). Zhu et al. investigated low-isotacticity PP in the SLS process and found that a PP with low crystallinity reduces the risk of shrinkage and part deformation. And this PP powder exhibits good SLS processability and sufficient mechanical strength [114].

Syndiotactic PP (sPP) differs from iPP in having a lower crystallinity and distinct crystallization kinetics. Cholewa et al. synthesized sPP and used it as a powder material for PBF technology. The sPP particles exhibit processable coalescence during laser sintering. With a melt viscosity intermediate between that of iPP and CoPP, sPP enabled the fabrication of dense parts with low porosity [115].

**Figure 5:**
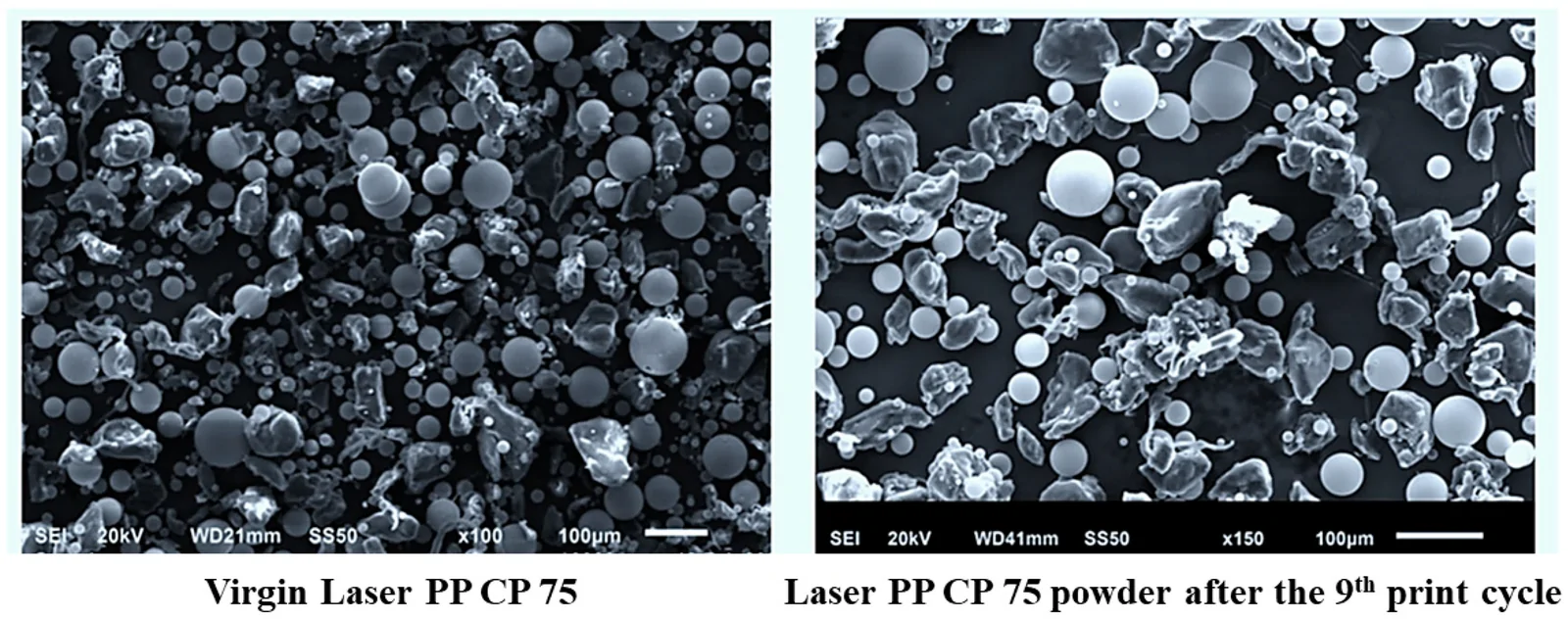
Powder morphology for Laser PP CP 75. Reprinted with permission from Ref. [72], copyright 2022 MDPI.

In order to reduce the cost, PP has also been recycled, especially Laser PP CP 75 polypropylene powder from Diamond Plastics GmbH [72]. Mwania et al. investigated Laser PP CP 75 to confirm whether the powder is 100% reusable. As can be seen in Figure 5, after nine print cycles, the shape of the powders remained. Although the parts of the recycling powder had a higher tensile strength and a lower stiffness, Laser PP CP 75 produces significant errors in dimensional accuracy, which limits its commercial application.

In recent years, composite PP materials have become research focuses.

#### 3.2.3. Composite PP Materials

Various carbonaceous materials, including CB, graphene, and CNTs, have been mixed with PP powders to improve the absorption of short-wavelength lasers. Luo et al. incorporated alkylating CB (aCB) into CoPP by melt extrusion after the alkylation disposal of CB through a Friedel–Crafts reaction. Compared with conventional CB/PP powders, aCB/PP powders exhibit superior rheological properties, a deeper laser penetration depth, and more stable laser absorptance. Furthermore, parts sintered using a diode laser and aCB/PP powders demonstrate fewer structural defects and reduced material degradation compared with those produced from CB/PP powders [116].

Shen et al. prepared a 3D conductive network of CB/graphene nanosheets/PP composites by the SLS method. The conductivity of the composites was eight orders of magnitude higher than that of pure PP [117]. Li et al. used another ternary composite of CNTs/mesocarbon/PP microbeads to improve conductivity. When the volume proportion of CNTs exceeded 45%, the composites could not be sintered. The conductivity of the composites increased with more conductive fillers, while the bending strength and hydrophobicity were weakened [118].

In order to enhance its thermal stability and mechanical strength, some mineral materials have been mixed with PP. Tan et al. studied several preparation methods, such as melt mechanical mixing, extrusion combined with cryogenic grinding, surface coating, and TIPS, to prepare montmorillonite (OMMT)/PP composites. They found that both mechanical mixing and surface coating are low-cost but exhibit a limited improvement in thermal stability. Melt extrusion combined with cryogenic grinding is considered the most balanced method, as it enabled uniform dispersion of OMMT particles in PP and significantly enhanced thermal stability. The TIPS method provided a wide sintering window and slower crystallization rate than other methods. The near-spherical OMMT/PP composites fabricated by TIPS have suitable particle size for the SLS process [119]. Unlike OMMT/PP composites, zeolite/PP composites are used to fabricate macroporous materials with higher adsorption capacity [120].

PEEK is also a common polymeric material for PBF processes.

### 3.3. PEEK

PA has limited applicability in high-temperature environments due to its relatively low Tm and glass transition temperature. Consequently, PEEK has been an alternative material for PA with a higher Tm, excellent biocompatibility, good chemical resistance, outstanding flame retardancy, lightweight properties, non-toxicity, and superior mechanical strength. These attributes make PEEK suitable for applications in the biomedical, automotive, and aerospace industries. However, the widespread adoption of PEEK is hindered by its low surface energy and chemical inertness, which result in poor adhesion to most substrates [121]. To address this challenge, various surface modification technologies, such as plasma treatment and chemical reaction, have been employed to improve its adhesive properties. Similar to PA, the performance and application of PEEK are affected by crystallization, aging, and sintering conditions.

#### 3.3.1. Crystallization of PEEK

The crystallinity of PEEK is a critical determinant of the mechanical strength of sintered products. Chen et al. investigated the quasi-static and non-isothermal crystallization kinetics of PEEK during the SLS process. Their study revealed that a mixture of 2D lamellar and 3D spherulitic crystallization formed at a theoretical Tb of 321 °C for PEEK. However, in practical applications, Tb is maintained at approximately 332 °C. A higher isothermal temperature is beneficial for improving mechanical strength and increasing crystallite thickness [122].

Research on PEEK crystallinity in fused filament fabrication (FFF) offers valuable insights into PBF technology. Collinson et al. observed that increased crystallite density at the welding interface during FFF leads to the formation of a rigid, immobile crystalline surface layer which consequently compromises mechanical strength. To mitigate this issue and promote the formation of amorphous PEEK parts, they reduced the chamber temperature from 130 °C to 70 °C during printing [123]. Han et al. researched the dynamic crystallization of PEEK using a phase-evolution model. Their study revealed that continuously forming crystals may undergo potential plastic deformation as new crystal phases develop [124].

Natural PEEK exhibits poor laser light absorbance, so it is often mixed with CF, CNTs, or GF to enhance its laser absorbance. However, these fillers influence the crystallization behavior and morphology of the PEEK matrix. Zhong et al. investigated the interfacial crystallization of PEEK on the surface of CF modified with a sizing agent. They found that the sizing agent induced transcrystallinity (TC) on the CF surface. The denser lamellar crystal structure of the TC layer improves interfacial properties, including tensile strength and resistance to solvent erosion. This may be attributed to the proper concentration of the sizing agent, which fills the voids generated by the high viscosity of molten PEEK [125].

Yang et al. proposed that the sizing agent and cooling rate primarily influence the nucleation process of PEEK crystallization. Specifically, a high cooling rate and the removal of the sizing agent promote nucleation, thereby increasing nucleus density. These factors also facilitate the transformation of crystal morphology from spherulitic to TC, which improves interfacial shear strength [126]. Meanwhile, Li et al. used polyaryletherketone (PAEK) with molecular weights ranging from 2000 to 8000 as sizing agents to regulate composite crystallization. They found that while the molecular weight of PAEK had no significant effect on interfacial shear strength, it was directly proportional to infiltration enhancement [127].

PEEK composites have garnered research attention due to their ability to modulate crystallization behavior and improve the practical application of PEEK. At present, CF/PEEK is the most prevalent composite material used in PBF. However, its widespread adoption is constrained by poor flowability. To solve this problem, Tang et al. used nanoscale SiO_2_ as a flow agent. They found that a SiO_2_ weight fraction below 0.6% effectively improved flowability, while further addition exceeding this threshold reduced the mechanical strength of the sintered composites [128]. In addition, CF/PEEK can be used for implant–abutment complexes. In a comparative study involving titanium, zirconia, CF/PEEK, glass-fiber-reinforced PEEK (GF/PEEK), and polyetherketoneketone (PEKK) acting as sintering materials, Elkersh et al. identified zirconia as the optimal material for high-density bone applications, while CF/PEEK emerged as the preferred choice for low-density bone structures [129].

Besides CF, hydroxyapatite (HA) and bioactive glass (BG) have also been used to fabricate PEEK composites for biomedical applications [130,131,132,133,134]. Bioactive HA has been successfully incorporated into PEEK matrices to enhance the performance of bone scaffolds. Tan et al. fabricated scaffolds with a predetermined internal architecture and highly porous scaffolds by controlling SLS sintering parameters [130]. Dey et al. found that the melting temperature and its holding time, cooling rate, and HA particle content affected HA/PEEK degradation, heterogeneous nucleation, and crystal growth [131]. Furthermore, Senra et al. introduced ZnO into HA/PEEK composites to tailor crystallization without compromising crystalline structure. ZnO can influence crystallization behaviors, thermal stability and antimicrobial activity. Notably, the addition of 2.5 wt% flower-like ZnO balanced the crystallization kinetics and material performance by improving the chain mobility [132].

The bioactivity-enhancing effect of hydroxyapatite (HA) is limited because only a small fraction acts on the surface. To address the inherent biological inertness and poor processability of PEEK, Wang et al. fabricated BG/PEEK composites with a wider sintering window, superior bioactivity, and higher thermal conductivity than pure PEEK [133]. Controlling crystallization and optimizing composite formulation are crucial for PEEK applications, alongside careful consideration of aging and sintering parameters.

#### 3.3.2. Aging of PEEK

Although PEEK is a high-performance engineering polymer with excellent thermal stability, it is still susceptible to aging, which leads to crosslinking and solidification. This process results in increased tensile strength and storage modulus, but decreased elongation. Guo et al. investigated PEEK aging by changing the gas type (air, nitrogen, and argon) and holding time (0–8 h) at the optimal Tb. They found that thermal aging caused chain crosslinking regardless of the gas type, whereas chain breakage only occurred in air. A reduction in the hardness of aged PEEK was observed in nitrogen after a holding time of 4 h. Thus, argon is recommended as the protective gas. The change in gas type does not alter the fact that aged PEEK exhibits a narrower particle size distribution, improved flowability, higher bulk density, and more regular microstructures. In detail, PEEK was partially decomposed and aged to a higher degree in air than in nitrogen and argon. So, the PEEK aged in air could not be reused and it was difficult to sinter it at the commercial high temperature of 330 °C. Moreover, the aged PEEK decomposed severely at a higher laser power and was unmelted when the laser power was lower. In addition, the flowability of the aged PEEK was affected by the balance between spherical and flaky surface morphologies [134].

Ahmad et al. investigated the aging behavior of PEEK under thermo-oxidative and humid conditions. They found that aging increased tensile strength and storage modulus, accompanied by decreased elongation at break and dielectric loss of PEEK [135]. In contrast, Miura et al. proposed that accelerated aging degrades the mechanical properties of PEEK by inducing defects in the crystal structure [136].

Besides crystallization and aging, sintering parameters are also crucial for the application of PEEK in PBF technology.

#### 3.3.3. Sintering Parameters

The key sintering parameters for PEEK include the laser power density, Tb, and sintering temperature. Wang et al. investigated the effects of varying laser power densities and sintering modes on the warpage and tensile properties of PEEK parts. Their results indicated that at a laser power density of 0.5 J·mm^−2^, warping deformation was inevitable under both low-power, slow-sweep and high-power, fast-sweep sintering modes, while an optimal laser power density of 0.2 J·mm^−2^ yielded superior flatness and tensile properties when using the low-power, slow-sweep mode [137].

Unlike Wang’s findings, Eren utilized a multi-physics finite element analysis model for PEEK and determined that the optimal sintering profile is achieved at a laser power density of 0.03 J·mm^−2^. He also considered that laser scanning speed was also important, as it governs heat accumulation and thermal gradients [138].

As mentioned above, PEEK composites have been widely used in PBF, and their sintering parameters differ from those of pure PEEK. Hu et al. adopted MH to sinter CNT/PEEK composites. In their process, PEEK particles were mixed with CNTs and subjected to ball milling to fabricate CNT/PEEK composites, then consolidated into dense samples under compressive stress. Since CNTs have a high microwave heating efficiency, the content of CNTs is a key factor in the MH process. The sintered CNT/PEEK composites demonstrated excellent electrical conductivity and achieved a tensile strength of 96.5 MPa [139].

Zhang et al. investigated the influence of processing parameters such as paving velocity, layer thickness, and fiber length on the mechanical performance and microstructure of sintered CF/PEEK composites. They found a non-monotonic relationship between paving speed and failure strength. Additionally, a lower layer thickness resulted in more aligned fibers than a higher layer thickness. Longer fibers contributed to higher failure strength than shorter ones, which can be attributed to enhanced alignment along the powder spreading direction [140]. Other factors, such as ambient humidity and sintering temperature, can also affect the sintering process [141,142].

In addition, the recycled Laser PP CP 75 polypropylene powder from Diamond Plastics GmbH was researched by Yan et al. They found that a lower preheating temperature (305 °C) decreased the process difficulty. And the recycled CF/PEEK parts showed smaller shrinkage and warpage than pure PEEK [143].

Moreover, the main sintering method for PEEK is SLS. PEEK’s high melting point, high melt viscosity and sensitive crystallization behavior constrain its application in HSS, MJF and MH methods.

### 3.4. TPU

Unlike PA, PP, and PEEK, TPU is a typical elastic material used in PBF technology. It can be reshaped following SLS processing and exhibits excellent mechanical performance. TPU has been widely used in footwear, films, rollers, and various automotive components. However, a significant challenge of TPU is its tendency to melt primarily at particle contact points during the SLS process. This leads to the formation of numerous microvoids. These voids compromise surface quality and contribute to surface contamination [144,145]. Thus, TPU composite materials have recently emerged as a research focus.

Various TPU composites have been developed to meet diverse application requirements. Zhang et al. used TPU/lithium chloride composites, assisted by the SLS method, to fabricate bio-inspired hierarchical porous cones, which achieved high-performance water harvesting performance. The cones enhanced moisture capture and storage and minimized clumping and leakage. And the CNT coating on the cones significantly increased evaporation efficiency [146].

Zhan et al. prepared three composites, TiO_2_/TPU, SiO_2_/TPU, and SiO_2_/TiO_2_/TPU, which exhibit self-cleaning properties and improved thermal stability. The particle sizes of TPU, TiO_2_, and SiO_2_ ranged from 20 to 80 μm, 10 to 50 μm, and 20 to 40 μm, respectively. They found that only when the particle sizes of TiO_2_, SiO_2_, and TPU were comparable did the addition of TiO_2_ and SiO_2_ fillers have a negligible impact on the fluidity of the composites. The self-cleaning characteristic was attributed to the low surface wettability of SiO_2_ and the surface roughness formed by unmelted TiO_2_ and SiO_2_ fillers. In addition, they incorporated a waterborne polyurethane emulsion modified with (3-(2-aminoethyl) aminopropyl) trimethoxysilane into SiO_2_/TPU to achieve materials with further improved self-cleaning performance and thermal stability [147,148].

CNTs serve as effective fillers to improve the electrical or thermal conductivity of TPU. For instance, Zhuang et al. fabricated pressure sensors using CNT/TPU composites via the SLS process. They dispersed CNTs in a TPU matrix through strong acid oxidation and ball milling, and then conducted orthogonal experiments to investigate the relationship between piezoresistive sensitivity, density, dimensional precision, laser power, layer thickness, and scan spacing. The resulting CNT/TPU sensor demonstrated a wide pressure detection range, high sensitivity, fast response time, and excellent stability and durability for human foot pressure monitoring [149]. Li et al. used multi-walled carbon nanotube (MWCNT)/TPU composites to prepare flex sensors by the SLS method, inverted-mold grooving, and ultrasonic cavitation-enabled treatment. The resulting sensors with hierarchical structures were capable of defecting or monitoring compression, curvature changes, and structural bending [150].

Besides the composites mentioned above, TPU can achieve conductive and hydrophobic properties through the incorporation of CNTs, zirconium sulfate (Zr(SO_4_)_2_), and aluminum tannate via SLS. Insulating properties can be realized by sintering glass microballoon/TPU composites. Furthermore, coumarin/TPU and SAOED/TPU composites serve as luminescent materials after SLS processing [151,152,153]. Moreover, the problems of surface roughness and inferior mechanical properties in SLS-molded TPU parts can be solved by chemical polishing and a solvent-assisted dip-dyeing process [154,155].

In TPU sintering, SLS offers a moderately wide sintering window, with laser scanning being a well-established method for TPU powders. HSS utilizes infrared lamps to uniformly heat areas printed with absorbing ink, thereby providing a wider sintering window than SLS. In contrast, MJF fabricates parts by jetting fusing and detailing agents. Although MJF also employs infrared lamps to heat the entire printed area, it has a narrower sintering window than HSS, which is similar to or slightly tighter than that of SLS. Some other polymers have been investigated according to different application demands.

### 3.5. Other Polymers

According to different market demands, other polymers, including PS, PC, UHMWPE, EVA, and polylactic acid (PLA), have been adopted in PBF technology. These polymers have different characteristics (as shown in Table 5).

As an early-used powder material for the SLS method, PS suffers from significant shrinkage and deformation during the sintering process, which leads to products with limited mechanical strength. To mitigate the issues mentioned above, some reinforcing agents such as nano-Al_2_O_3_, various fibers, and other polymers have been incorporated into PS powders. The addition of nano-Al_2_O_3_ improves the tensile strength and notched impact strength of sintered PS products, providing both reinforcement and toughening effects. Furthermore, the incorporation of CF, GF, or aramid fiber (AF) can also enhance the flexural strength and tensile strength of PS parts. Similarly, blending PS with acrylonitrile-butadiene-styrene (ABS), polyethylene (PE), polymethyl methacrylate (PMMA), polyamide (PA), or styrene-butadiene-styrene block copolymer (SBS) contributes to enhance the flexural strength of the resulting PS parts. Concomitantly, these modifications also reduce surface shrinkage of PS during SLS treatment, thereby improving molding accuracy [3,156,157,158,159,160,161,162,163].

It is known that PC has high toughness, good thermal stability and flame resistance. Kloos et al. fabricated spherical semi-crystalline PC powders for the SLS process by the TIPS method, addressing the limitations of amorphous PC in this application [164].

UHMWPE possesses outstanding mechanical properties, excellent impact resistance, and superior wear resistance, which makes it a suitable material for PBF technology. However, processing UHMWPE via sintering is challenging due to its uneven shrinkage and narrow sintering window. In addition, the density of sintered UHMWPE parts correlates positively with laser power. The high melt viscosity of UHMWPE makes it difficult to flow, which can be mitigated by incorporating some additives such as low-molecular-weight polyethylene or nano-/microparticles [165,166]. For instance, Gusarov et al. introduced Al_2_O_3_ microparticles into UHMWPE to enhance the heat resistance, uniaxial tensile strength, and rheological properties of SLS-sintered parts. Although the addition of Al_2_O_3_ reduces shrinkage and warping, it concurrently compromises uniaxial tensile properties and ductility [167].

In PBF technology, the properties of EVA are influenced by the content of vinyl acetate (VA) in the polymer. An increase in VA content leads to a gradual decrease in crystallinity, hardness, rigidity, and heat distortion temperature. Conversely, impact resistance, flexibility, and thermal shrinkage exhibit an upward trend. Thus, adjusting the VA content enables the customization of EVA for diverse applications [168].

Different from many other polymers, PLA exhibits excellent biocompatibility and favorable mechanical properties, which facilitated its widespread application in the medical field. For example, Li et al. prepared porous bone scaffolds by sintering calcium silicate/PLA composites using SLS. The composite improves the compressive strength and modulus of the scaffolds [169]. Furthermore, Liu et al. studied the behavior of PLA during the SLS process through molecular dynamics simulations. They divided the sintering process into three distinct stages: (1) initial intermolecular attraction driven by van der Waals forces; (2) molecular diffusion and entanglement leading to form sintering necks; and (3) fusion at molecular interfaces, where hydrogen bonds and end groups promote molecular packing [170].

Beyond the aforementioned polymer powders, polyphenylene sulfide and polyethylene wax have also been applied in PBF technology. These materials are particularly suitable for fabricating components featuring thin walls and complex curved geometries [171,172].

**Table 5:** Comparison of the characteristics of PS, PC, UHMWPE, EVA, and PLA.

Polymer Type	Sintering Window (°C)	Key Advantages	Primary Processing Challenges	Target Applications	Reference
PS	(Near Tg) 80–105	Good abrasion and chemical resistance	Significant shrinkage and deformation	Deliver good dimensional accuracy	[170]
PC	Around 144	High toughness, good thermal stability, fine flame resistance	High porosity, sparse parts	Rapid prototyping	[161,170]
UHMWPE	117–141	Outstanding mechanical properties, excellent impact resistance, superior wear resistance	Uneven shrinkage, narrow sintering window	Sporting goods, medical artificial joints, bullet proof jackets	[163]
EVA	85–105	Good cushioning properties, lightness, flexibility	Content of vinyl acetate	Rubber, foam	[165]
PLA	170–180	Excellent biocompatibility, favorable mechanical properties	Slow degradation, inadequate cell adhesion	Scaffold	[166,167,171]

Note: PS, polystyrene; PC, polycarbonate; UHMWPE, ultra-high-molecular-weight polyethylene; EVA, ethylene vinyl acetate copolymer. PLA, polylactic acid.

## 4. Conclusions and Recommendations

Sintering methods and related materials are pivotal in PBF technology. This review has summarized the primary sintering methods, including SLS, HSS, MJF, and MH. The characteristics of these methods are analyzed as follows: (1) The SLS method offers advantages in cost-effectiveness, process feasibility, and the ability to fabricate complex geometries without support structures; however, it is prone to shrinkage, warping, and deformation of polymer powders during sintering. (2) HSS/MJF uses infrared (IR) irradiation energy sources instead of laser beams, which reduces operational costs. Among these methods for polymers, SLS is the most widely adopted sintering method for PBF technology.

Besides sintering methods, polymer powders constitute a critical determinant in PBF technology. Polymers compatible with these processes include PA, PP, PEEK, and TPU, as well as PS, PC, and UHMWPE. Each material has its unique characteristics: (1) PA is a biocompatible polymer characterized by high tensile strength, superior impact resistance, excellent processability, low moisture absorption, and rapid melting kinetics. Particle size distribution, crystallization behavior, aging, composite formulations, and the optimization of sintering parameters are important for PA sintering by the SLS method. (2) PP powders have some advantages, including excellent chemical resistance, low density, and a relatively low processing temperature. For PP, polymerization type, crystallization behavior, and compositing with carbonaceous materials (CB, graphene, or CNTs) to improve laser absorption will influence its performance. (3) PEEK has numerous benefits, such as high biocompatibility, robust chemical resistance, superior flame retardancy, a lightweight nature, non-toxicity, and excellent mechanical strength. Crystallization, aging, and sintering parameters will affect sintered PEEK products. (4) TPU is an elastic material that can be readily reshaped using the aforementioned sintering methods.

The aforementioned research confirms that PBF technology plays a key role in AM fabrication. Despite rapid advancements in sintering techniques and polymer powder materials, challenges still remain, along with the demands of the smart manufacturing era. The following suggestions for future technological development are proposed: (1) The combination of different sintering methods is necessary. HSS/MJF has attracted significant research interest as it avoids laser sintering. Furthermore, combining SLS with the MH method may significantly reduce shrinkage, warping, and deformation. (2) New composite polymer powders will offer another means to overcome existing challenges. Extensive studies have explored polymer composites reinforced with metals, ceramics, or salts. (3) AI-assisted design will provide an opportunity for AM, particularly for PBF technology. As rapid advancements in AI usher in a new era of innovation across various fields, using AI-based analysis to identify the defect rate during high-speed thermal imaging, in-line closed-loop process monitoring, in situ or in-process feedback control, powder distribution, life cycle assessment, etc., will accelerate the development of PBF technology [172,173,174,175,176,177,178,179,180,181]. Nevertheless, substantial research and development remain essential to further refine sintering methods and enhance polymer powder materials.

## Figures and Tables

**Table 1:** Comparison of SLS, HSS, MJF, MH, LAPS (Large-Area Projection Sintering), SIS (Selective Inhibition Sintering), ProFusion, and SPJ (Single-Pass Jetting).

Type	Heat Source	Recoating Mechanism	Deposition Agent	Building Rate	Commercial Status
SLS	Laser	Selectively sintering	-	Fast	Fully commercialized
HSS	Infrared light	Selectively sintering	Absorbing ink	Fast	Fully commercialized
MJF	Infrared light	Selectively sintering	Fusing agent, detailing agent	Fast	Fully commercialized
MH	Microwaves	Uniform bulk sintering	-	-	Limited commercialization
LAPS	Visible light	Selectively thermally fuse	-	Comparable to MJF	Laboratory
SIS	Infrared light	Selectively inhibition	Detailing agent	Slower than MJF	Limited commercialization
ProFusion	Nearly one million diode lasers	Melting, layer by layer	Absorbing ink	Faster than conventional AM method	Pre-commercial
SPJ	Infrared light	Need a post-processing	Binder	Significantly accelerating process speed	Fully commercialized for metal mass production

**Table 2:** Different PA particle sizes and synthesis methods.

Type	Synthesis Method	Particle Size (μm)	Shape	Cost	Energy Consumption	References
CNF-PA12	Mechanical crushing	−50	Non-uniform, angular	Lower than CNT	Reduced	[40]
PA1212	Solvent precipitation	20–80	Spherical	Low cost	-	[41]
PA6-PU	Suspension polymerization	−35	Microspheres	Lower than TPU	-	[42]
PA12	TIPS	−55.2	Spherical	Low cost	Reduced	[36]
PA11	TIPS + liquid–liquid phase separation	−50.3	Coarse surface	Low cost	Reduced	[43,44]

Note: PA, polyamide; CNF, carbon nanofiber; CNT, carbon nanotube; PU, polyurethane.

**Table 3:** Effect of PA aging on the characteristics of products fabricated by SLS technology.

PA	Atmosphere	Aging	Build Cycles	Particle Size Distribution	Crystallinity	Young’s Modulus	Tensile Strength	Strain at Break	Porosity	Reference
12	N	M	2–10	No	↓	↓	↓	↓	-	[54]
12	N	M	9	No	No/↑	No/↓	↑/↓	↑	↑	[37]
12	A	O	-	-	↓	↑	↓	↓	-	[56]
12	A	O	-	-	↓	↑	↑	↓	-	[57]
12	N	M	4	-	No	-	-	-	-	[58]
12	N	M	-	-	↑	-	-	-	-	[59]
12	N	M	1	↑	↓	-	-	-	-	[60]
12	N	M	-	↑	↓	↓	↓	↑	-	[61]
12	N	M	7	-	↑	↓	↓	No	↑	[62]
12	N	M/O	3	-	↑	-	-	-	-	[63]
6/12	N	M	>5	↑/No	No	-	-	-	-	[64]
12	N	M	9	No	-	↑/↓	↑/↓	↑/↓	↑	[65]
12	N	M	>5	-	-	↑	↓	↓	-	[51]
12	N	M	6	No	↓	No	No	-	↑	[52]
12	N	M	8	No	-	-	-	-	-	[53]
12	N	M	5	-	↑	-	-	-	↑	[66]
12	N	M/O	5	-	-	↓	↓	↑/↓	-	[67]
11	N	M	1	↑	↑	-	↓	No	↑	[68]
6	N	M	7	-	↑/↓	↓	↓	↓	↑	[55]

Note: N, nitrogen; A, air; M, machine; O, oven; No, no change or no data reported; ↑, increasing; ↓, decreasing; -, no test.

**Table 4:** Comparison of the properties of iPP, CoPP, and PA12 powders [106].

	iPP	CoPP	PA12
Hausner ratio	1.28	1.22	1.13
Sa (μm)	48	52	30
Rc (μm)	186 ± 3.9	210 ± 2.0	124 ± 1.7
Rsm (μm)	452 ± 14.3	508 ± 9.9	380 ± 7.7
Sintering window (°C)	26.3	2.2	25.8
Melting range (°C)	21.5	51.3	13.8
Stable sintering region (°C)	172–271	150–298	198–360
Degree of crystallization (%)	47.7	28.1	43.4
Viscosity at (Tm ± 25) °C (Pa·s)	237	14,813	~400 [111]

Note: Sa, average surface roughness; Rc, mean height; Rsm, mean width; Tm, melting temperature; iPP, isotactic PP; CoPP, copolymer PP.

## Data Availability

No new data were created or analyzed in this study. Data sharing is not applicable to this article.

## Abbreviations

The following abbreviations are used in this manuscript:

AM	Additive manufacturing
PBF	Powder bed fusion
PA	Polyamide
PP	Polypropylene
PEEK	Polyether ether ketone
TPU	Thermoplastic polyurethane
FDM	Fused deposition modeling
SLS	Selective laser sintering
MH	Microwave heating
HSS	High-speed sintering
MJF	Multi-jet fusion
3D	Three-dimensional
PS	Polystyrene
PC	Polycarbonate
UHMWPE	Ultra-high-molecular-weight polyethylene
EVA	Ethylene vinyl acetate copolymer
TIPS	Thermally induced phase separation
DMAC	N,N-dimethylacetamide
PEG	Polyethylene glycol
DSC	Differential scanning calorimetry
CNP	Carbon nanoparticle
CNT	Carbon nanotube
CF	Carbon fiber
GF	Glass fiber
GB	Glass bead
LDH	Layered double hydroxide
APP	Polyphosphate
PER	Pentaerythritol
MOF	Metal–organic framework
BN	Boron nitride
iPP	Isotactic PP
CoPP	Copolymer PP
Sa	Surface roughness
Rc	Mean height
Rsm	Mean width
Tb	Bed temperature
sPP	Syndiotactic PP
CB	Carbon black
aCB	Alkylating CB
OMMT	Montmorillonite
TC	Transcrystallinity
PEKK	Polyetherketoneketone
HA	Hydroxyapatiteor
BG	Bioactive glass
MWCNT	Multi-walled carbon nanotube
Zr(SO4)2	Zirconium sulfate
PLA	Polylactic acid
AF	Aramid fiber
ABS	Acrylonitrile-butadiene-styrene
PE	Polyethylene
PMMA	Polymethyl methacrylate
SBS	Styrene-butadiene-styrene
VA	Vinyl acetate
AI	Artificial intelligence
